# Immediate changes in electroencephalography activity in individuals with nonspecific chronic low back pain after cranial osteopathic manipulative treatment: study protocol of a randomized, controlled crossover trial

**DOI:** 10.1186/s12906-015-0732-2

**Published:** 2015-07-13

**Authors:** Wagner Rodrigues Martins, Leonardo Rios Diniz, Juscelino Castro Blasczyk, Karina Ferreira Lagoa, Sérgio Thomaz, Marcia Elisabeth Rodrigues, Ricardo Jacó de Oliveira, Ana Clara Bonini-Rocha

**Affiliations:** College of Physical Therapy, University of Brasilia, Brasilia, DF Brasil; Brazilian Institute of Osteopathic, Rio de Janeiro, RJ Brazil; Faculty of Physical Education, University of Brasilia, Brasilia, DF Brazil

**Keywords:** Electroencephalography, Low back pain, Osteopathic manipulative treatment

## Abstract

**Background:**

Osteopathic medicine is based on a diagnostic and therapeutic system to treat tissue mobility/ motility dysfunctions in general, using different approaches (depending on the target tissue) known as osteopathic manipulative treatment. Among the available techniques those ones addressed to the cranial field are the most questioned because of the lack of scientific evidence; but the compression of the 4th ventricle technique has been largely studied in clinical trials. Studies have shown that the technique may affect both central and autonomous nervous system, modulating some reflexes (Traube-Hering baro signal), and modifying brain cortex electrical activity through central sensitization in subjects with chronic low back pain. Thus, investigators hypothesize that the compression of the 4th ventricle may modulate peak alpha frequency (eletroencephalographic assessment) and promote physical relaxation in subjects in vigil.

**Methods/Design:**

A randomized, controlled crossover trial with blinded assessor was designed to test the hypothesis. A total of 81 participants will be assigned to three treatment conditions, with seven days of washout: (I) compression of the 4th ventricle; (II) sham compression of the fourth ventricle; (III) control (no intervention). The (I) power amplitude and the (II) frequencies of the dominant peak in the alpha band will be the primary outcome measures of the study. All participants will be recruited at the Outpatient Rehabilitation Service of the University Hospital of Brasília – University of Brasília. All the electroencephalographic exams will be conducted by a blinded assessor.

**Discussion:**

The investigators hypothesize that patients with chronic low back pain submitted to the technique would have the peak alpha frequency modulated and, thus, would experience physical relaxation.

**Trial registration:**

NCT02111382

## Background

Osteopathic Medicine (OM) is based on a diagnostic and therapeutical system to treat tissue mobility dysfunctions in general [1]. The Osteopathic Manipulative Treatment (OMT) uses different techniques according to the dysfunctional tissues. The World Health Organization considers OM as complementary or alternative medicine, and it is based on concepts and unique approaches that enable the self-healing and self-regulating process within the body [2, 3]. This increasing interest in osteopathic medicine may reflect the reality of the society because patients turn to complementary or alternative treatments whereas the conventional treatments seem to fail in producing the desired outcome, or to produce side effects [4]. In the United States, the overall expenditure for complementary and alternative medicine is in the tens of billions of dollars per year [4].

Among the available OMT techniques, those addressed to the cranial field are the most questioned because of the lack of scientific evidence. A systematic review of 7 randomized controlled trials revealed studies heterogeneity, studies with poor methodology and not enough data to draw a conclusion about Osteopathy in the Cranial Field (OCF). One of the keystones of OCF teaching and practice is the presence of Primary Respiratory Mechanism (PRM) – which is partially represented by the movement of the cranial bones [5]. So, much discussion remains on the hands ability to perceive PRM through the obliterated sutures of the skull in adults, besides the lack of evidence to support them [6, 7].

One of the most studied OCF techniques is the Compression of the 4th Ventricle (CV4) - the osteopath exerts a compression force on the lateral angles of the squama occipitalis. The CV4 technique affects both central and autonomous nervous system [8], assessed by change in the blood flow and in the tissue oxygen saturation [9, 10]. Another way to measure the effects of the CV4 technique is the brain cortex electrical activity by electroencephalography (EEG), which is a validated method to support the results [11–14]. Current literature has only 2 crossover studies with healthy participants: the work of Cutler et al. [15] showed a decrease in sleep latency; and Miana et al. [16] showed an increase in the absolute power of alpha frequency. The EEG exam is useful to detect changes in the central processing of pain [17] and in patients with chronic pain, one study showed increased power amplitude in alpha frequency bands and a shift towards lower frequencies of the dominant peak [18]. These outcomes are important to develop new trials on diagnosis and therapeutics because of the increasing number of subjects with chronic pain in world [19, 20].

Therefore, this study aims to assess brain cortex electrical activity (alpha band frequency) in individuals with nonspecific chronic low back pain submitted to CV4 technique. The investigators hypothesize that patients with chronic low back pain submitted to the technique would have the peak alpha frequency (PAF) modulated and, thus, would experience physical relaxation.

## Methods and design

### Design and setting

This is a randomized, controlled crossover trial.

**Fig. 1:**
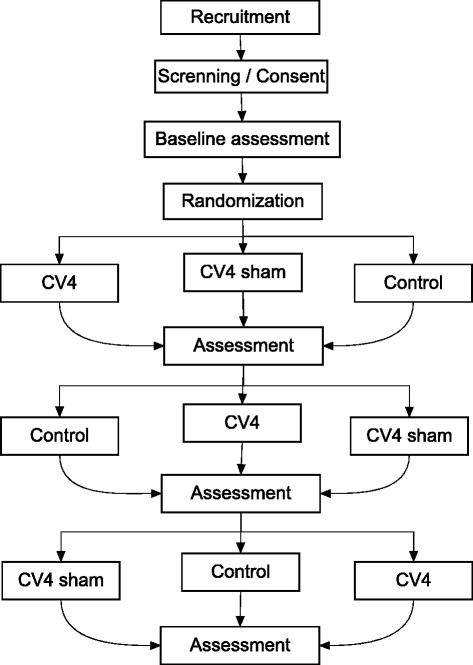
Trial flow chart showing cross-over design and assessment points

The participants will be recruited from the outpatient rehabilitation service of the University Hospital of Brasília from August 2015 to August 2017 through the media (daily papers, local newspapers, and public newsletters) at the hospital, and on the University of Brasilia website. The eligible participants will be randomly allocated in the three groups of study with seven days of washout. The participants will be given a written consent form before the start of the study, and they also will verbally consent the trial. Figure 1 summarizes the study planning.

The participants will be instructed to keep their sleeping and eating habits, as well as their daily living activities. They also will be advised not seek any other therapies, like massaging, physiotherapy, stretching and yoga to prevent any intervening factors during the trial period.

### Inclusion criteria

Participants over 18 years old with self-reported chronic low back pain will be recruited from the University Hospital of Brasilia. To ensure the inclusion criteria, an orthopedic physician will do the assessments all individuals who has interested in the study. Participants with chronic, recurrent, or long-lasting pain on musculoskeletal system for at least 6 months will meet the definition of chronic low back pain and, so, will be eligible to the study. To inclusion in the study the participants needs to presents: (I) a primary back pain form (i.e. progressive degenerative joint disease that result in biomechanical stresses affecting articular and extra-articular tissue) diagnosed by the physician according clinical and radiological findings; (II) only low back pain, to the Quebec Task Force [21]; and (III) absence of mental impairment determined by obtaining a score of 27 on the Mini Mental State Examination (MMSE) [22].

### Exclusion criteria

Participants suffering wiil be excluded if they present: (I) secondary chronic low back pain (ankylosing spondylitis, infections, malignancy, nephrolithiasis, pyelonephritis, aortic aneurysm, disease of the pelvic organs and gastrointestinal tract); (II) fever, chills, sweating, relate of weight loss superior of 5 % in the last 2 months without a clear cause, asthenia, anorexia; (III) cardiovascular and respiratory symptoms such as chest pain, dyspnea and cough; (IV) headache, dizziness, lightheadedness, fainting, tremors, dysarthria and aphasia; (V) surgery on the spine; (VI) diagnosis of any neurological, cardiac, respiratory and rheumatic disease; (VII) regular use of drugs with effects on the central nervous system (stimulants or depressors of CNS, examples: anxiolytics, anti-depressives, neuromodulators, etc.); (VIII) previous manual therapy treatment before data collection (in a four-eight weeks period of time); (IX) signs of nerve root involviment.

### Participants randomization and allocation

After participants selection, randomization will be performed using a computerized random number generator through the stratified simple randomization method of a software program (Excel, Microsoft Office 2008) for sequence generation. Sequentially numbered, opaque, sealed assignment envelopes will be delivered at the Hospital and the researcher will open one envelope in front of the participant; each envelope contains a concealed allocation number.

The three researchers performing the interventions will not have access to the order of allocation file. The allocation electronic file will be password-protected and only the fourth researcher will have the password to access it (ST).

### Intervention

All participants will receive three interventions at the Outpatient Rehabilitation Service of the University Hospital of Brasília (room F49, blue hall), respecting a 7-days washout interval and the same appointment time, which may be scheduled between 8 and 11 a.m.

#### CV4 technique

**Fig. 2:**
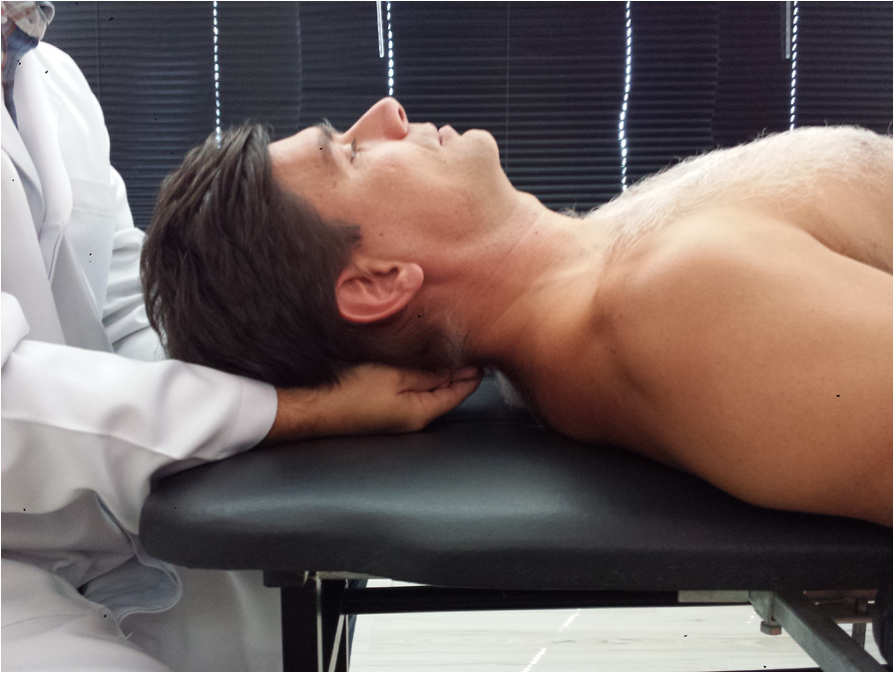
Picture of the CV4 technique

The participant will be in supine position during the whole procedure. The practitioner sits behind the participant’s head, contacts participant’s lateral protuberances of the squama occipitalis (medially to the occipitomastoid suture) with the thenar eminence of both hands. When the practitioner feels the PRM (a pulsating rhythm caused by liquor production and reabsorption), the flexion phase will be resisted and the extension phase exaggerated. The compressive force is held until the PRM stops (event known as ‘Still point’) [23] (10) (9). The compression will be held until the practitioner feels PRM gradual return; the compression will be slowly diminished, and then, the practitioner remove the hands from the occipital bone, laying the participant’s head on the table. Figure 2 shows the maneuver.

The flexion phase of the occipital bone comprises the perception of a centrifugal motion of the squama occipitalis and the flattening of its convexity. The extension phase comprises a centripetal motion of the squama occipitalis and an increase of its convexity. Thus, the practitioner intends to exaggerate the convexity of the squama occipitalis.

The CV4 technique will be performed by an osteopath D.O. - member of the Brazilian Register of Osteopaths.

#### CV4 sham

**Fig. 3:**
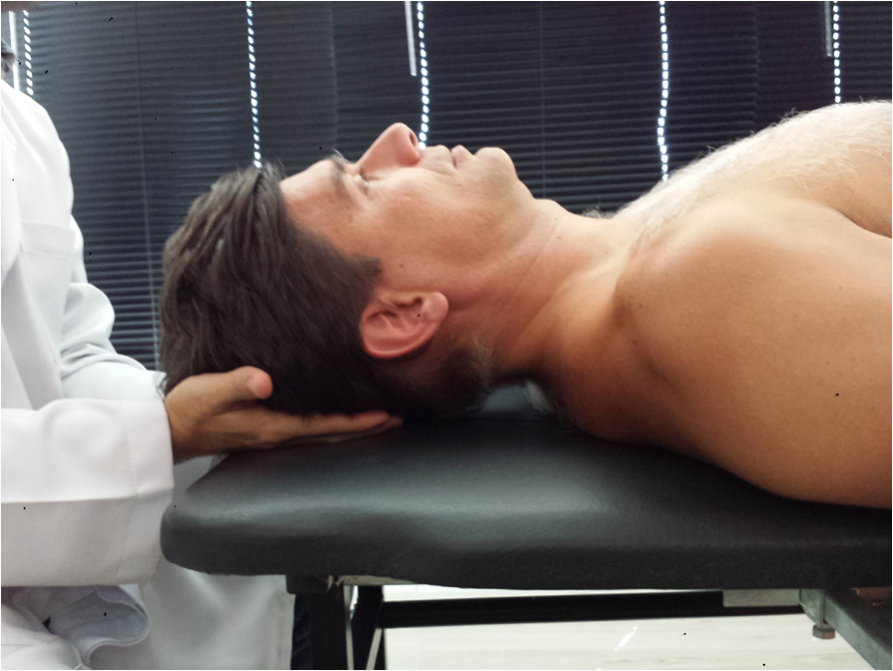
Picture of the sham technique

The participant will be in supine position and the practitioner will be seated behind the participant’s head. The practitioner places the fingers (2nd to 5th) under the occipital bone, touching only the squama occipitalis (Fig. 3). The practitioner will hold this position for the same time the CV4 duration, and after this period the participant’s head will be placed on the table.

The CV4 sham will be performed by a registered physiotherapist (ACBR).

#### Control

The participants will be in supine position for the same time the CV4 duration without any visual or verbal contact. A third researcher (a 5th-year osteopathic medicine student) will conduct this phase (KFL).

### Measurements

#### Primary outcome measurement

The mean change in peak alpha frequency (PAF) will be the primary outcome measure to assess the effects of CV4 technique on nonspecific chronic low back pain. PAF is a measure derived from EEG analysis, and it is defined by two parameters: (1) its occurrence on the frequency axis; and 2) its amplitude on the power density axis. The participants assessment (EEG) and the results statistical analysis will conducted by a blinded researcher (WRM).

#### Secondary outcome measurement

Secondary outcomes include the mean change in pain intensity (0–10 numeric pain ranting scale) before and immediately after each protocol section. The measurement (pre and post intervention) will be conduced by a researcher blinded to patient allocation (WRM).

#### EEG recording and analysis

The EEG record will be done before and immediately after each protocol section.

**Fig. 4:**
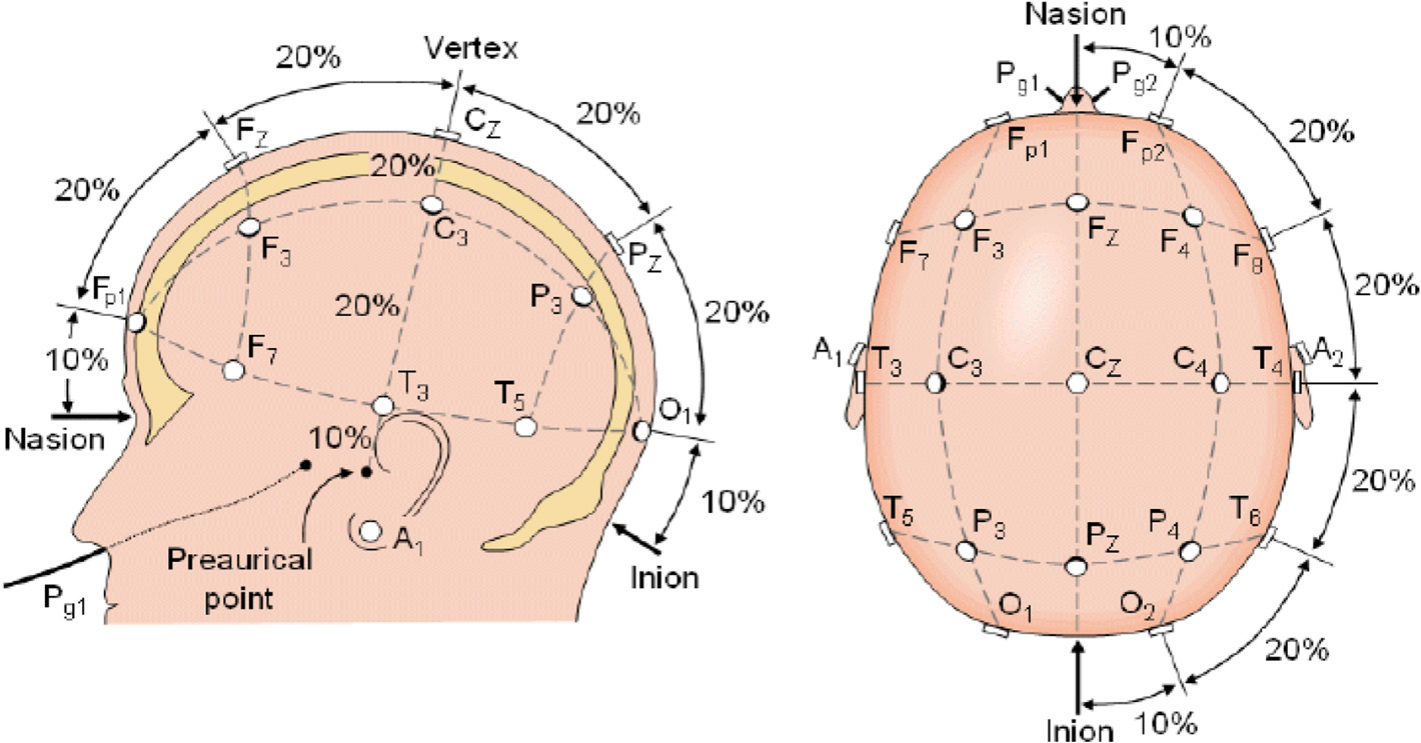
International 10–20 system [28]

EEG bipolar data will be collected from 26 scalp electrodes (Brain Wave II EEG, Inc., São Paulo, São Paulo) placed according to the International 10–20 system (Fp1, Fp2, F7, F3, Fz, F4, F8, FC3, Fcz, FC4, T3, C3, Cz, C4, T4, CP3, Cpz, CP4, T5, P3, Pz, P4, T6, O1, Oz, O2) [24, 25]. This system aims to standardize the electrodes arrangement in specific areas of the scalp surface regardless the size of the skull (Fig. 4). Electrooculogram data will be recorded from electrodes placed above and below the left eye orbit and at the lateral canthus of both eyes.

The EEG will be recorded with the eyes closed and opened, and each recording will last 2 min. During the exam, the participants will be in seated position (16). The participants will be given only two verbal commands: to open and close their eyes.

A computerized Fourier analysis of the EEG waves will be made, using the Brain Wave Analyzer Software. The signal will be sampled at 500Hz rate, and filtered with a 1–50 Hz band-pass filter. The signal will be filtered to display alpha frequency (8–13 Hz). Electrode impedance will be kept below 5KΩ, and the ground electrode placed at the Fpz location.

### Statistical analysis and sample size

#### Baseline and outcome data

The baseline characteristics will be shown as the mean ± SD for continuous data (i.e., participant’s age and duration of back pain), and n (%) for categorical data (i.e., gender). To analyze the baseline characteristics, we will perform a One Way analysis of variance, or a Kruskal-Wallis test for continuous data if the data are normally distributed. Shapiro-Wilk test will be used to test data normality. The primary and secondary (EEG and pain) outcomes will be analyzed by 3 × 2 mixed-effects model (group [CV4 - CV 4 sham - Control] x time [pre and post]) to repeated data measures for each continuous outcome variable, we will also assesses if the allocation order will interfere in results, for thus we’ll analyze the order of allocation of CV4 (1^st^, 2^nd^ or 3^rd^) x pre values (CV4 – CV4 sham – Control) at the 3^rd^ exposition, using a one-way Anova or Kruskal-Wallis test. When the analysis of variance indicates significant differences among groups, a post-hoc test (Bonferroni test) will be used to separate the differences between groups before and after the intervention period. Data will be analyzed using Prism 6 software for Mac OS X, and a significance level of *p* ≤ 0,05 will be considered to all variables.

#### Compliance

Participants will be considered dropped-out if they do not attend the second and/or third day of intervention.

#### Sample size

The sample size was calculated considering: (1) the two-way analysis of variance (ANOVA); [26] three groups; (3) type I error = 5 % (2-sided); (4) type II error = 20 %; (5) the power of the statistical test = 80 %; and (6) effect size = 20 %. The effect size was obtained considering: (I) the results from the pilot study of Miana et al. [16], which demonstrated a effect of 26 % (pre to post intervention) in the group that received the CV4 technique and (II) Cohen’s *d* which considers 20 % a small effect to calculate sample size and power analysis [27, 28]. Then, it was determined a total of 81 subjects. This calculation was performed in G Power 3 program, version for Mac OS X.

### Side effects

The participants will report every unexpected and unintentional response not related to CV4 technique at every visit.

It is important to establish that this study will not recruit participants suffering from any disease because of the nature of research and the strict exclusion criteria. Regarding CV4 technique, the side effects of cranial manipulation are rare, but described in patients with traumatic brain syndrome [29]. The CV4 technique provides a smooth and gentle manual contact, promoting relaxation and wellness.

### Participants protection and ethics

The protocol was developed according to the general ethical guidelines, such as Declaration of Helsinki and the new version (2012) of 196/96 resolution of the Brazilian National Health Council (National Research Ethics Committee) for regulatory guidelines for research involving human subjects. This research project and the informed consent were submitted and approved by the Ethics Committee of the Faculty of Health Sciences of University of Brasília. The statement of ethical approval had the code CAAE 27525814.1.0000.0030. For publication of images (Figs. 2 and 3) was obtained the consent of the participant in the first day of experiment.

## Discussion

The aim of the study is to assess the CV4 technique effects on nonspecific chronic low back pain measured by the mean change in the peak alpha frequency (PAF). For this purpose, it was designed a randomized, controlled crossover study with 81 participants.

The investigators hypothesize that CV4 technique can modulate brain cortex electrical activity measured by EEG mean change in the peak alpha frequency. Some studies have investigated CV4 effects on healthy individuals, and there is evidence of changes in the central pain processing – supported by increased power amplitude differences in the alpha band and a shift towards lower frequencies of the dominant peak, measured by resting state EEG; but there is no study on persistent nonspecific low back pain in the current literature.

Therefore, the positive outcomes that can be drawn from this study would support the use of the CV4 technique in designing a sustainable and cost-effective health service for people with chronic low back pain because of the positive effects on functional reorganization human cortex.
